# Sport anxiety and subjective happiness of college athletes: a self-determination theory perspective

**DOI:** 10.3389/fpsyg.2024.1400094

**Published:** 2024-07-24

**Authors:** Zhiwen Zhang, Xiaoyu Chen, Liyu Xu, Xiao Qin, Arsaythamby Veloo

**Affiliations:** ^1^Faculty of Sports and Health, Chongqing Electronic Information College, Chongqing, China; ^2^Faculty of Social Sciences & Liberal Arts, UCSI University, Kuala Lumpur, Malaysia; ^3^School of Foundation Courses, Chongqing Institute of Engineering, Chongqing, China; ^4^Hope College, Southwest Jiaotong University, Chengdu, China; ^5^School of Liberal Arts, Shinawatra University, Pathum Thani, Thailand; ^6^Faculty of Education & Liberal Studies, City University Malaysia, Kuala Lumpur, Malaysia; ^7^Guangxi Water Conservancy and Electric Power Vocational and Technical College, Nanning, China; ^8^School of Education and Modern Languages, University of Northern Malaysia, Sintok, Malaysia

**Keywords:** sport anxiety, subjective happiness, self-determination theory, college athletes, concentration disruption

## Abstract

**Introduction:**

Sport anxiety not only impacts the performance of college athletes but also influences their psychological well-being. The psychological well-being of sports students is crucial for both academic performance and competition, as they need to balance their academic performance with professional athletic training.

**Method:**

Based on self-determination theory, this study examines the relationship between various factors in sport anxiety (somatic anxiety, worry, and concentration disruption) and subjective happiness, as well as the mediating role of need satisfaction in this relationship. A total of 835 college athletic students participated in the study, completing the Sport Anxiety Scale-2, Basic Psychological Needs Satisfaction Scale-in General, and Subjective Happiness Scale.

**Results:**

An analysis of gender differences revealed that female participants scored significantly higher on somatic anxiety (*t* = −2.21, *df* = 833, *p* = 0.028, Cohen’s *d* = −0.155) and worry (*t* = −3.17, *df* = 833, *p* = 0.002, Cohen’s *d* = −0.223) compared to males. In the analysis by sport type, participants engaged in team sports scored significantly higher on somatic anxiety (*t* = 2.70, *df* = 833, *p* = 0.007, Cohen’s *d* = 0.187), Worry (*t* = 1.97, *df* = 833, *p* = 0.049, Cohen’s *d* = 0.136), and concentration disruption (*t* = 2.73, *df* = 833, *p* = 0.007, Cohen’s *d* = 0.189) than those in individual sports. Additionally, in the analysis by grade level, freshman college athletes exhibited significantly lower sport anxiety compared to sophomore athletes [*F*(4, 830) = 4.06, *p* = 0.003, $\eta_{p}^{2}$ =0.019]. The mediation analysis revealed that concentration disruption in sport anxiety is significantly and negatively related to subjective happiness. Additionally, need satisfaction (competence, autonomy, and relatedness) mediates the relationship between worry, as well as concentration disruption in sport anxiety and subjective happiness.

**Discussion:**

Future research should build on the current study by employing longitudinal designs and integrating multiple objective measures to further explore the relationship between sport anxiety and subjective happiness.

## Introduction

Mental health issues undoubtedly impact every aspect of our lives, permeating through work and studies. For college athletic students, the burden is twofold, requiring regular participation in practices and competitions while also attending classes and completing assignments. The NCAA Student-Athlete Well-Being Study, which investigated the mental health of student-athletes, revealed alarming trends (NCAA, 2022). According to the study, student-athletes with mental health problems are 1.5 to 2 times more likely to experience mental health issues compared to the pre-COVID-19 era. Significantly, higher rates of anxiety and mental exhaustion were observed in this population. In a recent study involving 615 athletic college students, it was found that 129 participants surpassed the average college student in both state and trait anxiety (Weber et al., 2023). Therefore, a study of the relationship between sport anxiety and subjective happiness in college athletes is necessary to identify potential protective factors and provide evidence for targeted mental health interventions.

### Anxiety and sport anxiety

Anxiety is a psychological state triggered by an anticipated threat or potential threat and is a normal part of the human experience. However, excessive anxiety can have a direct impact on our lives and learning and can even develop into a disorder (Gross and Hen, 2004). For example, previous research has found that individuals with high anxiety perform more poorly on decision-making tasks such as the Iowa Gambling Task compared to those with low anxiety (Miu et al., 2008). Meanwhile, a meta-analysis that included more than 100 studies found anxiety to be significantly and negatively correlated with academic performance (Seipp, 1991). There are various types of anxiety, with the most common being state and trait anxiety (Spielberger et al., 1971). State anxiety represents an emotional state characterized by feelings of tension, apprehension, and increased autonomic nervous system activity. In contrast, trait anxiety refers to enduring individual differences in anxiety tendencies that are integrated with personality traits. Also included in the spectrum of anxiety are social anxiety (Morrison and Heimberg, 2013; Askari and Zia-Tohidi, 2023), characterized by a fear of rejection and negative evaluation in social interactions, and sports anxiety (Smith et al., 1990, 2006), which manifests in the context of athletic competition.

Anxiety is one of the most commonly researched topics in sports psychology, not only because it affects an athlete’s mental health but also due to its direct impact on performance in both training and competition (Woodman and Hardy, 2003). In order to measure anxiety associated with sports activities more effectively, researchers have developed specialized tools designed for assessing sports-related anxiety. Examples of such tools include the Competitive State Anxiety Inventory-2 (Martens et al., 1990) and the Sport Anxiety Scale-2 (SAS-2) (Smith et al., 2006). Each scale has distinct characteristics; for instance, the CSAI-2 assesses cognitive anxiety, somatic anxiety, and self-confidence, while the SAS-2 targets sport-specific trait anxiety, encompassing three factors: somatic anxiety, worry, and concentration disruption.

### Anxiety and subjective well-being

Subjective well-being refers to an individual’s subjective assessment of their own life, encompassing both cognitive and emotional evaluations of personal or general events. It involves judgments of satisfaction with various aspects of life and incorporates what people commonly refer to as happiness and life satisfaction (Diener et al., 2003). Anxiety, characterized as a state of negative affect—opposite to happiness—undoubtedly influences subjective well-being (McNiel et al., 2010). For instance, individuals with General Anxiety Disorder commonly experience intense feelings of worry and struggle to manage them, leading to distress (Wittchen and Hoyer, 2001). The Cognitive-Behavioral Model of General Anxiety Disorder suggests that these individuals adopt negative thought patterns and distorted beliefs, interpret life events negatively, diminish overall life satisfaction, and ultimately impact subjective well-being (Dugas et al., 2005). Previous research has established significant negative correlations between both trait and social anxiety and life satisfaction as well as positive affect in well-being (De Castella et al., 2014).

Despite the mental health benefits of participating in sports, athletes become more susceptible to anxiety disorders and depression as the pressure to compete intensifies (Rice et al., 2016). Student-athletes are more susceptible to mental health problems, which may be related to their athletic identity and dual career motivation. Sports students must consider how to balance their roles and identities as both students and athletes. This balance, often referred to as “athletic identity,” pertains to the extent to which an individual identifies with the role of an athlete (Brewer et al., 1993). Previous research has identified significant differences in athletic identity among college athletes based on age and level of competition. Specifically, younger athletes tend to have higher identity, while athletes competing at higher levels exhibit more pronounced identity (Lupo et al., 2017a). In addition, a student-athlete’s social identity can predict their psychosocial adjustment over time (Parker et al., 2021). Notably, athletes with a high athletic identity reported higher levels of depressive symptoms during the COVID-19 pandemic compared to those with medium or low athletic identity (Antoniak et al., 2022). Additionally, student-athletes possess dual career motivations: academic motivation and athletic (or sport) motivation. These motivations can, respectively, impact their academic performance and psychological well-being (Gaston-Gayles, 2004; Stenling et al., 2015). Previous studies among Italian student-athletes have identified significant differences in academic and sport motivation based on gender, sport type, and competition level (Lupo et al., 2017b).

As mentioned earlier, anxiety not only influences the athletic performance of a college athlete but also has a significant impact on their mental health. Prior meta-analytic studies have revealed a positive correlation between anxiety and burnout (Koutsimani et al., 2019). Furthermore, a study on a sample of young athletes discovered a correlation between athlete trait anxiety and burnout, with cognitive reappraisal identified as a mediating factor (Gomes et al., 2017). In addition, previous studies have identified sport anxiety as a key variable in predicting dysfunctional coping behaviors among college students in sports (Contreras et al., 2023). Given the high prevalence of anxiety among college athletic students (Weber et al., 2023), it is crucial to investigate the relationship of sport anxiety and subjective happiness, explore potential protective factors, and consequently offer targeted guidance for mental health and happiness interventions in this population (Egan, 2019). Furthermore, prior research has not explored the distinct contributions of various factors of sports anxiety on subjective happiness. For instance, how do somatic anxiety, worry, and concentration disruption differ in influencing subjective happiness?

### Self-determination theory and subjective well-being

Over the past few decades, Self-Determination Theory (SDT) has explored the connection between motivation and subjective well-being (Ryan and Deci, 2002). SDT is a macro-theory encompassing human motivation, personality development, and well-being. Specifically, SDT centers on self-determined behavior and the social and cultural conditioning that supports it (Deci and Ryan, 2012). SDT proposes a set of fundamental and universal psychological needs—autonomy, competence, and relatedness. These needs are deemed essential for an individual’s psychological growth, integrity, and well-being, transcending cultural contexts and stages of development. When these three needs are nurtured and fulfilled within the social environment, individuals experience heightened vitality, self-motivation, and overall well-being. Conversely, thwarting or undermining these basic needs leads to diminished self-motivation and reduced well-being (Deci and Ryan, 2012).

Researchers have also initiated inquiries into the relationship between anxiety and the need satisfaction, exploring how this connection influences various other variables. For example, previous research has explored the connection between social physique anxiety and psychological needs related to physical activity. It has revealed that social physique anxiety directly impacts need satisfaction and, through psychological needs, indirectly influences physical behavior (Brunet and Sabiston, 2009). In a recent large-sample study, the effects of negative affect on psychological need satisfaction and subjective well-being were investigated. The results indicated that negative affect had a negative impact on all three types of need satisfaction in SDT, ultimately influencing participants’ life satisfaction (Šakan et al., 2020). At the same time, the three need satisfactions of SDT were identified as partial mediators between negative affect and life satisfaction.

### The present study

This study aims to investigate the relationship between sport anxiety and subjective happiness among college athletes, as well as to explore the mediating role of need satisfaction in this relationship from the perspective of SDT. Drawing on the insights from prior research (Koutsimani et al., 2019; Contreras et al., 2023), we formulated our first hypothesis (H1) that college athletes’ sports anxiety is negatively associated with subjective happiness. In addition, based on previous findings (Brunet and Sabiston, 2009; Šakan et al., 2020), we anticipated that the three need satisfactions in SDT would act as mediators in the relationship between sport anxiety and subjective happiness (H2). Finally, previous research has found that some demographic variables of participants also influence sport anxiety (Ramis et al., 2015; Marín-González et al., 2022; Martínez-Gallego et al., 2022). For example, significant differences in sport anxiety have been found by gender (male vs. female) and by type of sport (individual vs. team) (Ramis et al., 2015). Additionally, other studies have found significant differences in sport anxiety across age, education level, and competitive level (Marín-González et al., 2022). Therefore, given the sample size of the current study, we aim to further compare differences in key variables (e.g., sport anxiety) across gender, sport type, grade level, and competitive level.

## Methods

### Participants

The current study was conducted using the online questionnaire platform, where participants accessed the questionnaire by scanning a QR code with their mobile phones. A total of 951 collegiate sports athletes voluntarily took part in the study. These participants completed the survey as part of their coursework and received course credit in return. To maintain data quality, we incorporated appropriate quality-check questions within the questionnaire. For example, one question asked, ‘What color is a ripe banana (red, yellow, blue, or black)?’ Participants who chose incorrectly (the correct answer being ‘yellow’) were excluded from the final data analysis. Additionally, responses with low completion times (less than 30 s) were also excluded to ensure the participants’ commitment to their answers. Consequently, the final data analysis included 835 sports athletes’ students. There were 494 male and 341 female participants, with a mean age of 20.63 years and a standard deviation of 3.32 years. Among the participants, 475 were freshmen, 219 were sophomores, 37 were juniors, 8 were seniors, and 96 were graduate students. They trained in various sports, including basketball (128), football (109), volleyball (34), table tennis (205), badminton (94), Wushu (21), track and field (41), water sports (15), dance (23), gymnastics (10), tai chi (15), tennis (5), and other sports (135). We categorized these participants based on the type of sport they played, with 409 engaged in individual sports (e.g., swimming, and martial arts) and 426 involved in team sports (e.g., basketball and tennis). Additionally, 621 of these participants have competed at the university level, 82 at the city level, 91 at the provincial level, 32 at the national level, and 9 at the world level. The study protocol received approval from the local school ethics committee.

### Measures

#### The sport anxiety scale-2 (SAS-2)

The SAS-2 is a 15-item questionnaire designed to measure the levels of anxiety experienced by athletes before and during competition (Smith et al., 2006). The scale comprises three factors: somatic anxiety, worry, and concentration disruption, each consisting of five items. Participants in this study responded using a four-point Likert scale, where 1 indicated “not at all” and 4 indicated “very much.” Higher scores on the scale indicate greater levels of sports anxiety experienced either before or during competition. The SAS-2 has demonstrated strong psychometric properties in various languages, including the Chinese version, which was employed in the present study. Our research utilized the same Chinese version of the SAS-2 as utilized in previous study (Zhang et al., 2023). Regarding the reliability of the SAS-2 in our study, we calculated Cronbach’s alpha coefficients, which yielded values of 0.86 for somatic anxiety, 0.87 for worry, and 0.85 for concentration disruption. The overall reliability coefficient for the entire scale was 0.94.

#### Basic psychological need satisfaction scale-in general (BPNSS-G)

The BPNSS-G is designed to assess the overall fulfillment of individuals’ needs in various aspects of life (Ryan and Deci, 2002). It specifically evaluates three fundamental needs: competence, autonomy, and relatedness, comprising a total of 21 items. Participants responded to these items using a 7-point Likert scale, where 1 denoted “not at all true” and 7 represented “very true.” For our research, we adopted the Chinese version of the BPNSS-G (Liu et al., 2013), which was translated and validated. This Chinese version consists of 19 items and has shown good structure validity and robust reliability in previous research (Liu et al., 2013). In our present study, we computed Cronbach’s alpha coefficients for the three factors of the BPNSS-G. These coefficients were 0.76 for competence, 0.79 for autonomy, and 0.73 for relatedness. Furthermore, the reliability coefficient for the total scale was 0.84.

#### Subjective happiness scale (SHS)

The SHS comprises four items that assess individuals’ agreement with statements regarding their well-being and life satisfaction (Lyubomirsky and Lepper, 1999). The SHS is a widely employed self-report measure that provides a straightforward yet valid assessment of subjective happiness (Spagnoli et al., 2012; Extremera and Fernández-Berrocal, 2014). In our current study, we utilized the Chinese version of the SHS, which has demonstrated robust psychometric properties in a Chinese context (Nan et al., 2014). The Cronbach’s alpha reliability coefficient for the SHS in our study was 0.76.

### Procedure

Participants completed the questionnaire anonymously using their mobile phones by scanning a QR code during their physical education (PE) sessions. Data for this study were collected through multiple visits to various universities.

### Statistical analysis

The study employed SPSS 22.0 (IBM Corp., United States) and JASP version 0.18.03 (JASP Team, 2024) for statistical analyses, maintaining a significance level (alpha) of 0.05. Specifically, the data were first tested for common method bias, followed by descriptive and correlation analyses between variables. Next, independent samples *t*-tests and one-way ANOVA were used to test for differences in demographic variables on sport anxiety, need satisfaction, and subjective happiness. Finally, mediation analysis tests were conducted using JASP version 0.18.03.

## Results

### Test for common method bias

In the present study, we employed two strategies to address potential common method bias concerns. First, our measurement instrument incorporated diverse scale formats (Podsakoff et al., 2003). Second, we conducted statistical analyses to evaluate the extent of common method bias (Podsakoff et al., 2003). Specifically, we performed a Harman one-factor test on the three principal variables in our study—Sport anxiety, Basic Psychological Need Satisfaction, and Subjective happiness. The exploratory factor analysis, employing an unrotated factor solution, revealed the presence of five factors. Notably, the first factor accounted for the largest covariance at 26.01%. This finding suggests that common method bias is unlikely to significantly impact the study’s results as a contaminant.

### Descriptive and correlation analysis

We computed the mean, standard deviation, and Pearson correlation coefficient for each variable in our study. The detailed results are presented in Table 1.

**Table 1:** Descriptive statistics and correlations.

Variable	*M*	*SD*	1	2	3	4	5	6	7
1. Somatic anxiety	9.07	3.03	–						
2. Worry	10.55	3.42	0.75^**^	–					
3. Concentration disruption	8.55	2.91	0.79^**^	0.69^**^	–				
4. Competence	27.08	4.72	−0.27^**^	−0.28^**^	−0.32^**^	–			
5. Autonomy	26.02	4.29	−0.28^**^	−0.30^**^	−0.32^**^	0.73^**^	–		
6. Relatedness	32.98	6.24	−0.21^**^	−0.19^**^	−0.28^**^	0.73^**^	0.68^**^	–	
7. Subjective Happiness	19.36	4.12	−0.26^**^	−0.23^**^	−0.28^**^	0.45^**^	0.43^**^	0.44^**^	–

*N* = 835, **p < 0.01. **Correlation is statistically significant at the 0.01 level (two tailed). M, mean; SD, standard deviation.

The correlation results indicated significant negative correlations between all three factors of the SAS-2 and the need for competence, autonomy, and relatedness, as well as a significant negative correlation with subjective happiness. Additionally, the need for competence, autonomy, and relatedness exhibited significant positive correlations with subjective happiness.

### Differences in demographic variables in sport anxiety, need satisfaction, and subjective happiness

To better understand the effects of participant demographic variables on sport anxiety, need satisfaction, and subjective happiness, we conducted independent samples t-tests for different genders and types of sport, followed by one-way analyses of variance for participants in different grades and levels of competition.

As shown in Table 2, female participants scored significantly higher than males on somatic anxiety (*t* = −2.21, *df* = 833, *p* = 0.028, Cohen’s *d* = −0.155) and worry (*t* = −3.17, *df* = 833, *p* = 0.002, Cohen’s *d* = −0.223) in sport anxiety. In addition, the differences between participants of different genders on the three needs satisfaction as well as subjective happiness were not significant.

**Table 2:** Results of independent samples *t*-tests on key variables by gender.

Variables	Male	Female	*t*	*p*	Cohen’s *d*
Somatic anxiety	8.88 ± 3.00	9.35 ± 3.06	−2.21	0.028	−0.155
Worry	10.25 ± 3.42	11.00 ± 3.38	−3.17	0.002	−0.223
Concentration disruption	8.52 ± 2.98	8.60 ± 2.82	−0.38	0.707	−0.026
Competence	27.05 ± 4.77	27.11 ± 4.66	−0.20	0.843	−0.014
Autonomy	25.84 ± 4.24	26.28 ± 4.35	−1.46	0.146	−0.103
Relatedness	32.63 ± 6.06	33.48 ± 6.48	−1.94	0.053	−0.136
Subjective happiness	19.28 ± 4.19	19.47 ± 4.02	−0.67	0.502	−0.047

Comparison of participants practicing different types of sports revealed that those practicing individual sports scored significantly higher on somatic anxiety (*t* = 2.70, *df* = 833, *p* = 0.007, Cohen’s *d* = 0.187), worry (*t* = 1.97, *df* = 833, *p* = 0.049, Cohen’s *d* = 0.136), and concentration disruption (*t* = 2.73, *df* = 833, *p* = 0.007, Cohen’s *d* = 0.189) than those practicing team sports. Similarly, participants practicing different sports did not differ significantly in need satisfaction and subjective happiness (as shown in Table 3).

**Table 3:** Results of independent samples *t*-tests on key variables by type of sport.

Variables	Individual	Team	*t*	*p*	Cohen’s *d*
Somatic anxiety	9.36 ± 3.02	8.79 ± 3.03	2.70	0.007	0.187
Worry	10.79 ± 3.40	10.33 ± 3.43	1.97	0.049	0.136
Concentration disruption	8.83 ± 3.05	8.28 ± 2.75	2.73	0.007	0.189
Competence	27.00 ± 4.61	27.15 ± 4.83	−0.44	0.662	−0.030
Autonomy	25.89 ± 4.37	26.15 ± 4.21	−0.89	0.376	−0.061
Relatedness	32.80 ± 6.18	33.16 ± 6.31	−0.83	0.405	−0.058
Subjective happiness	19.10 ± 4.14	19.60 ± 4.09	−1.75	0.081	−0.121

The analysis revealed significant differences in somatic anxiety by grade level (Table 4), *F*(4, 830) = 4.06, *p* = 0.003, $\eta_{p}^{2}$ =0.019, *Post hoc* comparisons indicated that freshmen scored significantly lower than sophomores (*p* = 0.003). Additionally, there were significant differences in concentration disruption across grades, *F*(4, 830) = 5.09, *p* < 0.001, $\eta_{p}^{2}$ =0.024, with freshmen scoring significantly lower than sophomores (*p* = 0.003) and juniors (*p* = 0.024). However, the difference in worry across grades was not significant, *F*(4, 830) = 2.18, *p* = 0.069, $\eta_{p}^{2}$ =0.010. Finally, the differences in competence (*p* = 0.131), autonomy (*p* = 0.125), relatedness (*p* = 0.108), and subjective happiness (*p* = 0.085) across different grades were not significant.

**Table 4:** Descriptive data on sport anxiety, need satisfaction and subjective happiness of participants in different grades (*M ± SD*).

Variables	Freshmen	Sophomores	Juniors	Seniors	Graduate students
Somatic anxiety	8.81 ± 2.90	9.69 ± 3.30	9.65 ± 2.84	9.63 ± 3.16	8.69 ± 2.92
Worry	10.28 ± 3.35	11.06 ± 3.53	11.08 ± 3.39	10.38 ± 2.88	10.56 ± 3.46
Concentration disruption	8.26 ± 2.80	9.11 ± 2.96	9.76 ± 2.99	8.25 ± 3.24	8.28 ± 3.03
Competence	26.95 ± 4.49	26.90 ± 4.82	26.54 ± 4.52	27.50 ± 5.24	28.25 ± 5.49
Autonomy	26.32 ± 4.35	25.58 ± 4.10	25.35 ± 2.89	27.63 ± 4.44	25.70 ± 4.77
Relatedness	33.23 ± 6.25	33.28 ± 6.19	31.54 ± 6.56	34.88 ± 6.81	33.73 ± 6.05
Subjective happiness	19.60 ± 4.09	18.83 ± 4.15	18.54 ± 5.03	18.25 ± 1.58	19.76 ± 3.88

The only significant difference between participants at different levels of competition was in competence for general need satisfaction (Table 5), *F*(4, 830) = 2.51, *p* = 0.041, $\eta_{p}^{2}$ =0.012, *Post hoc* comparisons revealed that participants who competed at the university level had lower competence scores than those who competed at the provincial level (*p* = 0.069).

**Table 5:** Descriptive data on sport anxiety, general need satisfaction and subjective happiness of participants in different competition level (*M ± SD*).

Variables	University level	City level	Provincial level	National level	World level
Somatic anxiety	9.08 ± 3.04	9.16 ± 3.04	8.85 ± 3.10	9.41 ± 2.80	8.56 ± 3.05
Worry	10.53 ± 3.42	10.24 ± 3.31	10.56 ± 3.37	11.97 ± 3.70	9.67 ± 3.54
Concentration disruption	8.54 ± 2.84	8.51 ± 2.94	8.41 ± 3.24	9.21 ± 3.20	8.67 ± 3.28
Competence	26.79 ± 4.55	27.66 ± 4.75	28.22 ± 5.62	27.94 ± 4.97	26.89 ± 3.76
Autonomy	25.94 ± 4.26	26.17 ± 4.55	26.71 ± 4.33	25.66 ± 4.20	24.89 ± 3.14
Relatedness	32.74 ± 6.14	33.20 ± 6.20	33.87 ± 6.67	34.81 ± 7.35	32.22 ± 3.96
Subjective happiness	19.20 ± 4.15	19.38 ± 4.13	19.78 ± 3.63	20.75 ± 4.69	20.44 ± 3.61

### Mediation model analysis

To test the hypotheses, a mediation analysis was conducted with the three factors of sport anxiety (somatic anxiety, worry, and concentration disruption) as independent variables, the three needs of SDT as mediator variables, and subjective happiness as the dependent variable. Gender, sport type, grade, and competition level were included as control variables, and bootstrap resampling (5,000 samples) was applied.

**Figure 1:**
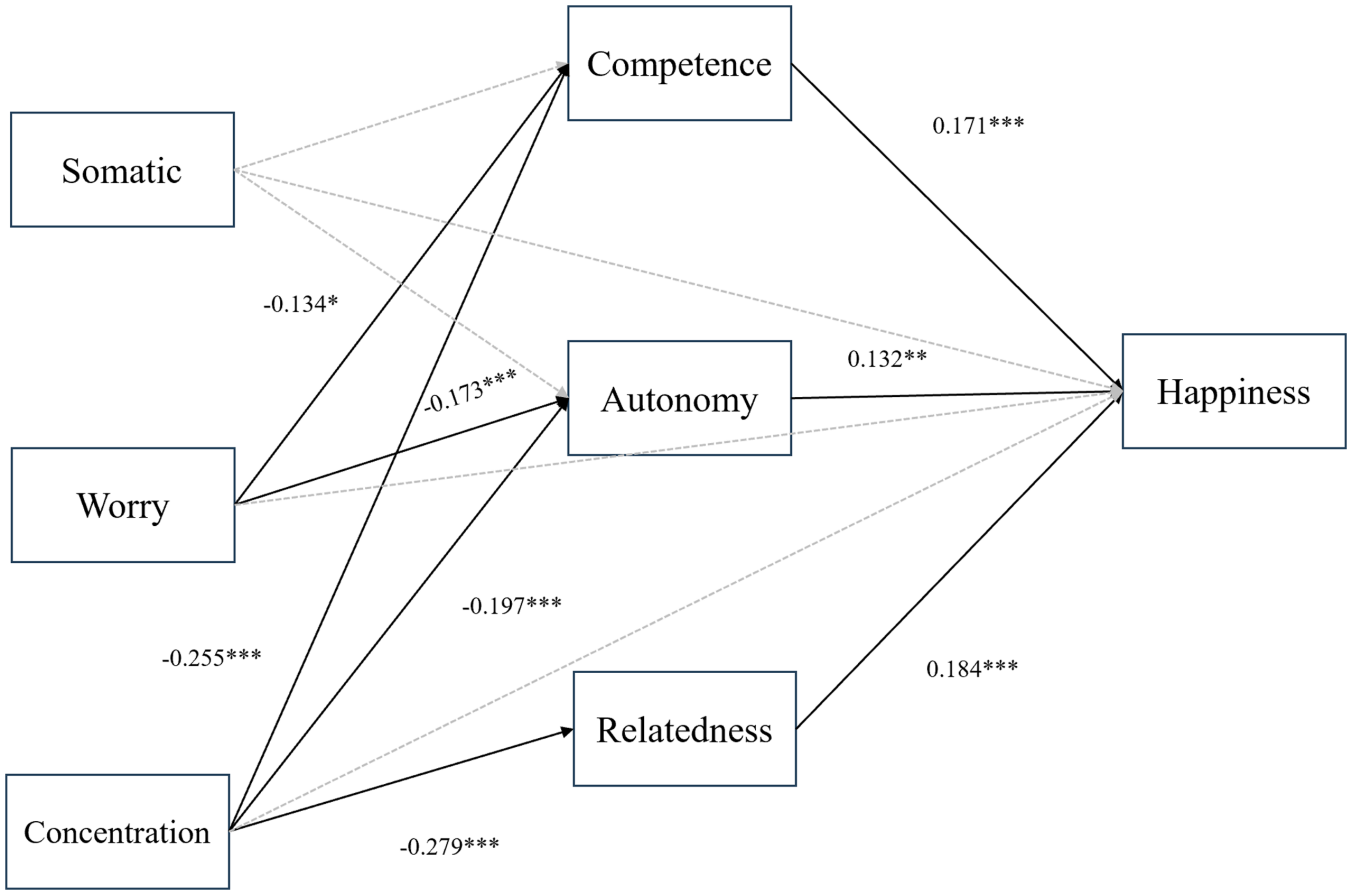
Mediation analysis path results. Solid lines indicate significant path effects, while gray dashed lines represent non-significant path effects. ***p < 0.001, **p < 0.01,*p < 0.05.

The results of the mediation analysis found that among the total effects, concentration disruption in sport anxiety (effect = −0.167, *SE* = 0.056, *p* = 0.003) was significantly negatively related to subjective happiness (Table 6). This result partially supports Hypothesis 1, indicating that only concentration disruption in sport anxiety is significantly negatively related to subjective happiness. In the indirect effects, we found that the three types of need satisfaction played a significant mediating role in the relationship between worry (effect = −0.051, *SE* = 0.023, *p* = 0.030) as well as concentration disruption (effect = −0.121, *SE* = 0.026, *p* < 0.001) in sport anxiety and subjective happiness, respectively. In this context, competence and autonomy need satisfaction mediate the relationship between worry and subjective happiness, while competence, autonomy, and relatedness mediate the relationship between concentration disruption and subjective happiness. This result also partially validates Hypothesis 2, which posits that the three need satisfactions mediate the relationship between sport anxiety (worry and concentration disruption) and subjective happiness (Figure 1).

**Table 6:** Effects of each path.

					95% Confidence interval	
Path	Estimate	*SE*	*z*-value	*p*	Lower	Upper
Direct effects						
Somatic – happiness	−0.098	0.055	−1.774	0.076	−0.206	0.008
Worry – happiness	<0.001	0.047	0.010	0.992	−0.093	0.089
Concentration – happiness	−0.046	0.051	−0.905	0.365	−0.151	0.055
Total indirect effects						
Somatic – happiness	−0.009	0.026	0.327	0.743	−0.042	0.056
Worry – happiness	−0.051	0.023	−2.169	0.030	−0.098	−0.007
Concentration – happiness	−0.121	0.026	−4.713	<0.001	−0.168	−0.077
Total effects						
Somatic – happiness	−0.089	0.061	1.462	0.144	−0.206	0.029
Worry – happiness	−0.050	0.052	−0.967	0.333	−0.149	0.045
Concentration – happiness	−0.167	0.056	−2.997	0.003	−0.276	−0.061

SE, std. error. Happiness, subjective happiness; Somatic, somatic anxiety; Concentration, concentration disruption.

## Discussion

Using the SDT framework, this study examined the relationship between sport anxiety and subjective happiness, as well as the mediating role of psychological need satisfaction, in a sample of college athletes. We first found that demographic variables affect sport anxiety, with significant differences observed between participants of different genders, types of sports, and grade levels. Subsequently, mediation analysis revealed that concentration disruption in sport anxiety was significantly and negatively related to subjective happiness. Additionally, the three need satisfactions from SDT mediated the relationship between sport anxiety (worry and concentration disruption) and subjective happiness. The results of our study not only uncovered the relationship between different factors of sport anxiety and subjective happiness but also revealed the mediating mechanisms in this relationship.

The first goal of our study was to investigate the relationship between sport anxiety and subjective happiness among college athletes. We found that worry and concentration disruption in sport anxiety were negatively correlated with subjective happiness, consistent with previous research findings (Gomes et al., 2017; Contreras et al., 2023). While earlier studies have primarily focused on anxiety’s effects on burnout and coping styles, our findings contribute to a deeper understanding of the relationship between sport anxiety and subjective happiness. Findings from the current study further elucidate the relationship between different factors of sport anxiety and subjective happiness. Specifically, concentration disruption in sport anxiety was negatively correlated with subjective happiness. Worry was significantly negatively correlated with subjective happiness indirectly through competence and autonomy in need satisfaction. No correlation was found between somatic anxiety and subjective happiness. In sport anxiety, concentration disruption measures difficulties related to concentration in competitive activities, somatic anxiety captures physiological responses like muscle tension, and worry reflects concerns about performance (Smith et al., 2006).

To delve deeper into the relationship between sport anxiety and subjective happiness, we investigated the roles played by the three needs in SDT. Our results found that concentration disruption in sport anxiety was negatively correlated with all three need satisfactions, while worry was negatively correlated with competence and autonomy in need satisfaction, aligning with previous research (Brunet and Sabiston, 2009; Šakan et al., 2020). In addition, we found that the three types of need satisfaction in SDT were positively related to subjective happiness. In the mediation analyses, the needs in SDT were identified as mediators of the relationship between the different factors of sport anxiety (worry and concentration disruption) and subjective happiness. This discovery echoes previous research indicating that negative affect influences life satisfaction through the satisfaction of the three needs in SDT (Šakan et al., 2020). Considering the mediating role of the three need satisfactions in SDT in the relationship between sport anxiety and subjective happiness, an intervention program aimed at increasing these need satisfactions could help reduce the negative effects of sport anxiety on college athletes.

In addition, we examined differences in some demographic variables on sport anxiety, need satisfaction, and subjective happiness. The results found that female college athletes scored significantly higher on the somatic anxiety and worry factors of sport anxiety than male college athletes, consistent with findings from previous studies (Ramis et al., 2015; Marín-González et al., 2022; Martínez-Gallego et al., 2022). Women are more likely to suffer from anxiety disorders, influenced by factors such as biological influences, temperament, stress and trauma, cognitive factors, and environmental factors (McLean and Anderson, 2009). Additionally, the results indicated that participants engaged in individual sport programs had significantly higher levels of anxiety compared to those involved in team sports, aligning with findings from previous studies (Ramis et al., 2015; Marín-González et al., 2022). It’s possible that anxiety and self-confidence among college athletes may not reliably predict performance when competing in team sports, as the collective efforts of other players can significantly influence game outcomes, potentially mitigating individual anxiety levels (Craft et al., 2003). Finally, we also found that college athletes in their freshman year had significantly lower anxiety levels than college athletes in their sophomore year. This result may be attributed to increased academic and competitive pressures experienced by athletes in higher grades, leading to higher anxiety levels.

### Limitations and future research

The current study is not without its limitations. First, the current study used a cross-sectional design to investigate the relationship between sport anxiety and subjective happiness, which prevents us from making inferences about the causal relationship between these variables. Therefore, future research should consider using a longitudinal design to investigate this relationship, allowing for more robust inferences about causality. Second, all measurement instruments utilized in this study were based on self-report, which may introduce biases into the results (e.g., social desirability), despite our efforts to mitigate this by implementing quality checks to exclude non-serious responses. Future studies could use multiple measures or sources of data to increase the reliability of the results. For example, incorporating physiological metrics such as galvanic skin response and cortisol levels to assess participants’ emotional and physiological arousal. Third, the study’s sample comprised athletes from a typical athletic university rather than elite athletes. It is known that elite-level athletes often encounter higher levels of stress related to their athletic commitments, experiencing it more frequently, intensely, and for longer durations than their lower-level counterparts (Arnold et al., 2016). Elite athletes also demonstrate increased susceptibility to depression and anxiety during times of heightened stress (Rice et al., 2016). Therefore, caution should be exercised when generalizing the findings of this study to professional elite athletes. Future research should prioritize the inclusion of diverse measures in studies involving elite athletes, thus offering a more comprehensive understanding of the relationship between anxiety and subjective well-being in this specific population. Such insights would be invaluable in tailoring effective mental health intervention programs for professional elite athletes.

Finally, another limitation of this study is that it did not take into account other important factors such as college athletes’ identity and motivation, their social support, and coping mechanisms. As mentioned in the introduction, student-athletes possess dual motivations related to their academic and athletic careers, as well as dual identities as students and athletes. It is important to note that both identity and motivation are associated with mental health. High levels of autonomy lead to increased self-confidence and reduced anxiety (Menegassi et al., 2018), while negative emotions related to exercise identity can affect both state and trait anxiety. Additionally, the level of trait anxiety decreases with increasing self-identity (Masten et al., 2006). Previous research has found that higher levels of satisfaction with social support from family and friends are associated with lower anxiety symptom scores (Sullivan et al., 2022). Therefore, future research should also measure identity and motivation as well as social support in college athletes, considering that these factors can vary greatly depending on demographic variables and can have a moderating role in the relationship between sports anxiety and subjective happiness (Lupo et al., 2017a,b).

## Conclusion

In conclusion, this study explored the relationship between sport anxiety and subjective happiness in college athletes, as well as the mediating role of need satisfaction. These findings suggest that concentration disruption in sport anxiety is directly related to subjective happiness. Additionally, the three types of need satisfaction in SDT play a mediating role in the relationship between sport anxiety and subjective happiness. Specifically, competence and autonomy in need satisfaction mediate the relationship between worry and subjective happiness in sport anxiety. Finally, there were significant differences in college athletes’ sport anxiety across gender, sport type, and grade level. The results of this study not only shed further light on the relationship between sport anxiety and subjective happiness but also provide new insights into mental health intervention programs and strategies in college physical education.

## Data availability statement

The raw data supporting the conclusions of this article will be made available by the authors, without undue reservation.

## Ethics statement

The studies involving humans were approved by School of Foundation Courses, Chongqing Institute of Engineering. The studies were conducted in accordance with the local legislation and institutional requirements. The participants provided their written informed consent to participate in this study.

## Author contributions

ZZ: Writing – review & editing, Writing – original draft, Methodology, Investigation, Formal analysis. XC: Writing – review & editing, Validation, Methodology. LX: Data curation, Writing – review & editing, Validation. XQ: Writing – review & editing, Validation, Supervision, Methodology. AV: Supervision, Methodology, Writing – review & editing, Validation.
